# Mapping Polymer Molecular Order in the SEM with Secondary Electron Hyperspectral Imaging

**DOI:** 10.1002/advs.201801752

**Published:** 2019-01-20

**Authors:** Robert C. Masters, Nicola Stehling, Kerry J. Abrams, Vikas Kumar, Martina Azzolini, Nicola M. Pugno, Maurizio Dapor, Andreas Huber, Philip Schäfer, David G. Lidzey, Cornelia Rodenburg

**Affiliations:** ^1^ Department of Materials Science and Engineering University of Sheffield Sheffield S1 3JD UK; ^2^ European Centre for Theoretical Studies in Nuclear Physics and Related Areas (ECT*‐FBK) and Trento Institute for Fundamental Physics and Applications (TIFPA‐INFN) Trento 38123 Italy; ^3^ Laboratory of Bio‐Inspired and Graphene Nanomechanics Department of Civil Environmental and Mechanical Engineering University of Trento Trento 38123 Italy; ^4^ Ket‐Lab Edoardo Amaldi Foundation Rome 00133 Italy; ^5^ School of Engineering and Materials Science Queen Mary University of London London E1 4NS UK; ^6^ Neaspec GmbH Bunsenstrasse 5 82152 Planegg Germany; ^7^ Department of Physics and Astronomy University of Sheffield Sheffield S3 7RH UK

**Keywords:** electron microscope, molecular order, organic electronics, polymer, secondary electron spectroscopy

## Abstract

Understanding nanoscale molecular order within organic electronic materials is a crucial factor in building better organic electronic devices. At present, techniques capable of imaging molecular order within a polymer are limited in resolution, accuracy, and accessibility. In this work, presented are secondary electron (SE) spectroscopy and secondary electron hyperspectral imaging, which make an exciting alternative approach to probing molecular ordering in poly(3‐hexylthiophene) (P3HT) with scanning electron microscope‐enabled resolution. It is demonstrated that the crystalline content of a P3HT film is reflected by its SE energy spectrum, both empirically and through correlation with nano‐Fourier‐transform infrared spectroscopy, an innovative technique for exploring nanoscale chemistry. The origin of SE spectral features is investigated using both experimental and modeling approaches, and it is found that the different electronic properties of amorphous and crystalline P3HT result in SE emission with different energy distributions. This effect is exploited by acquiring hyperspectral SE images of different P3HT films to explore localized molecular orientation. Machine learning techniques are used to accurately identify and map the crystalline content of the film, demonstrating the power of an exciting characterization technique.

## 1 Introduction

A growing body of recent work has established the modern field of secondary electron (SE) energy spectroscopy.^1^, ^2^, ^3^, ^4^, ^5^ This technique in the scanning electron microscope (SEM) has enabled fresh, exciting insights in to the properties of polymeric, organic, and biological materials by exploiting the relationship between the emitted SE energy distribution and various material properties. By performing energy‐selective detection of SEs in the SEM, techniques such as secondary electron hyperspectral imaging (SEHI) have been used to form images which can map nanoscale variations in chemistry or molecular ordering.^3^, ^4^

To date, studies applying SE energy spectroscopy principles have largely been based upon empirical relationships between the SE spectrum and sample features. We have demonstrated that such empirical studies are sufficient to underpin many powerful applications of SEHI. However, delivering fully on the potential of SEHI, as a characterization technique, requires a robust understanding of the nature of SE spectra and the ability to link spectral features with specific sample properties. Developing these links is a complex task.

SE emission in the SEM results from a “cascade” of electron–sample interactions initiated by the primary electron beam. SEs generated within the material may each interact multiple times with sample atoms, electrons, phonons, and trap sites.^6^ Every interaction can influence the angle and energy of an emitted SE, and as such, the energy distribution of emitted SEs results from a complex convolution of various material and electronic properties. Peaks in the SE spectrum have been linked with the energy levels of conduction band minima in graphite^7^, ^8^ as well as the nanoscale bonding structure in organic materials.^9^ The effect of sample doping on the spectrum shape is also well documented.^10^, ^11^ However, building a deeper understanding of the origin of SE spectral features will unlock new analytical capabilities, for example in using a sample's SE spectrum to probe and map its electronic or chemical properties directly.

In this work, we systematically investigate the sample properties that influence the SE spectrum of a polymer, and in doing so provide a powerful example of the potential capabilities of SEHI. We use the technique to investigate and map molecular ordering in poly(3‐hexylthiophene) (P3HT), a semicrystalline polymer with a range of organic electronic applications.^12^ Localized molecular ordering in a P3HT film is a crucial aspect determining its electronic properties,^13^, ^14^, ^15^, ^16^ as the electron transport through a bulk film is defined by the nature and interconnectivity of its crystalline domains.^17^ More broadly, the importance of localized molecular order is observed across many polymer science applications, from organic photovoltaics^18^ to drug‐delivery systems.^19^ By probing the nature of ordering with SEM‐level resolution, we demonstrate a compelling new characterization tool which can aid development and understanding of a range of new materials.

We forge links between the molecular order of P3HT films and the SE spectrum using both an experimental approach and an advanced Monte Carlo modeling technique.^2^ We identify the sample properties that have the strongest influence on the shape of the SE spectrum, and explore how this spectrum relates to molecular ordering. Further, we apply SEHI methods to map molecular ordering across P3HT samples for the first time. Hyperspectral SE maps of P3HT films are analyzed with advanced spectral decomposition methods, powered by machine‐learning, to map the relative fraction of amorphous and crystalline material across the surface of P3HT with <100 nm resolution. As a result, we demonstrate SEHI as a robust, analytical microscopy technique that can dramatically expand the capabilities of the modern‐day SEM.

## 2 Results and Discussion

### 2.1 Secondary Electron Energy Spectroscopy

**Figure 1:**
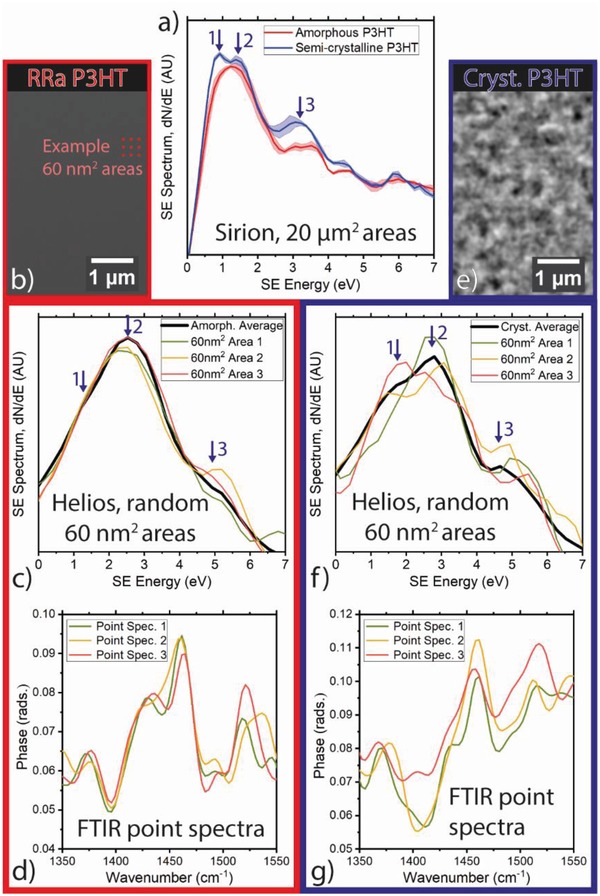
Secondary electron spectroscopy of P3HT, localized chemical variation. a) Compares low‐magnification SE spectra of amorphous and semicrystalline P3HT, measured from 20 µm^2^ areas in the FEI Sirion SEM. b–d) Consider localized variation in amorphous (regiorandom) P3HT films. b) Shows a conventional SEM image of the film, and c) shows SE spectra measured from 60 nm^2^ areas in the FEI Helios SEM. The scale of these measurement areas is depicted by red squares in (b). d) Shows nano‐FTIR point spectra measured from random areas on the film. e–g) Similarly consider localized variation in a semicrystalline P3HT film. e) Shows a SEM image of a semicrystalline film and f) shows SE spectra measured from random 60 nm^2^ areas of the film in the FEI Helios SEM. g) Shows nano‐FTIR point spectra measured from random areas of a semicrystalline P3HT film.

In **Figure** **1**, we present SE energy spectra measured from two different types of P3HT films, which we define as amorphous and semicrystalline. These are terms of convenience for this work, albeit not entirely accurate descriptors for the different types of film. The amorphous film was processed from regiorandom P3HT, whereby the random orientation of side‐chains acts to inhibit crystallite formation.^14^, ^20^ Regiorandom films are predominantly homogeneous and amorphous.^21^ In contrast, the semicrystalline film was processed from highly regioregular P3HT using a high‐boiling point solvent and subsequently treated with a thermal anneal. This encourages the formation of crystalline phases within the film alongside a considerable amorphous fraction.^17^, ^21^ Our full sample preparation methods are included in the Supporting Information for this work.

Secondary electron spectra measured from the different P3HT films in a FEI Sirion SEM are presented in Figure 1a. The experimental methods used to acquire and process these data can be found in the Supporting Information. The spectra in Figure 1a were acquired with a total dose of ≈3.4 × 10^13^ electrons cm^−2^. While the spectra have broadly the same form, some notable differences can be observed resulting from different levels of molecular ordering. First, around 1 eV, the semicrystalline P3HT sample is seen to have two peaks (peaks 1 and 2, denoted by arrows) where the amorphous P3HT sample has only one. Second, a higher energy peak around 3 eV (peak 3, denoted by an arrow) has higher intensity in the semicrystalline P3HT sample. We attribute these differences to the crystalline phases in the semicrystalline sample. These results were consistently reproduced from at least five different P3HT samples of each type measured over >1 year, with the shaded regions in Figure 1a demonstrating the standard error on spectra measured from three different areas of the same sample, spaced by ≈500 µm. With no significant location‐dependent variation observed in these data, we assume that wide field spectra are representative of the bulk properties of the sample.

### 2.2 Localized Variation in SE Spectra

To explore the contribution of localized molecular order in the SE spectrum emitted from P3HT, SE spectra were measured from different areas ≈60 nm^2^ in size, using a modern Helios Nanolab SEM. Due to the smaller measurement area, these spectra were acquired with a total electron dose of 2.16 × 10^15^ electrons cm^−2^, almost two magnitudes larger than that used to acquire SE spectra in Figure 1a. As such, SE spectra in Figure 1c (amorphous) and Figure 1f (semicrystalline) are susceptible to the effects of charging and electron beam damage, which results in these spectra having a slightly different form and peak positions in comparison to Figure 1a. However, the important molecular order related SE spectral features observed in Figure 1a are still present in Figure 1f (denoted by arrows). We assume these features are equivalent with those identified in Figure 1a and thus have the same origin.

Example SE spectra measured from 60 nm^2^ areas are presented in Figure 1c (amorphous) and Figure 1f (semicrystalline). Here, these small‐area spectra, plotted with colored lines, are compared with larger (≈2 µm^2^) measurement area spectra, plotted with a thicker black line. We observe that in Figure 1f, the localized variation in SE spectra is significant. Specifically, the relative intensity of peaks 1, 2, and 3 in Figure 1f appear to fluctuate across the sample surface. To separate the influence of molecular order on the SE spectra from any potential localized charging or shot noise effects on peak position or total spectrum intensity we compare the variations in relative intensities between peaks 1 and 2. For the semicrystalline sample, we find a standard deviation of ≈14% around the mean for ≈2500 spectra. This suggests that the SE transport and/or emission properties of the semicrystalline P3HT film are heterogeneous on the scale of tens of nanometers. In contrast the amorphous P3HT film is more homogeneous, as evidenced by the smaller variation in SE spectra in Figure 1c. The intensity of peak 1 relative to that of peak 2 for the same spectrum showed a standard deviation of ≈9% for this sample (≈2500 spectra). This greater homogeneity is in line with the lack of observable features in the SE image of this sample (Figure 1b). These observations suggest a link between molecular ordering and the fine structure in the SE spectra. Directly corroborating this link is difficult with established techniques. However, here we apply experimental and modeling techniques to further establish the connection. One established technique known to reflect local molecular order is Fourier‐transform infrared spectroscopy (FTIR).

### 2.3 Nano‐FTIR: Localized Chemical Variation

Nano‐FTIR enables the chemical properties of P3HT films to be analyzed on the nanoscale, allowing the correlation of localized variation in sample chemistry with variation in SE spectra (methods described in the Supporting Information). In Figure 1d,g, we compare nano‐FTIR spectra measured from different areas of semicrystalline and amorphous P3HT samples. Specifically we consider the symmetric C—C and antisymmetric C=C stretching modes measured here at 1460 and 1520 cm^−1^ respectively.^22^ The intensity ratio between the 1460 and 1520 cm^−1^ peaks, *I*_1460_/*I*_1520_, reflects the effective conjugation length of the film at the measured point^23^ and has been strongly linked to molecular ordering and electron mobility in the film.^23^, ^24^

In Figure 1g, significant variation in the different point spectra can be observed, reflecting variation in the localized chemistry of the semicrystalline film. The *I*_1460_/*I*_1520_ ratio of different point spectra is seen to fluctuate by ≈10% around the average reflecting localized variation in ordering and electron transport through the film. This correlates with the nature the narrow‐field SE spectra in Figure 1f.

Considering the amorphous P3HT film, we note that both the narrow‐field SE spectra (colored plots in Figure 1c) and nano‐FTIR point spectra (Figure 1d) demonstrate only small variations across the sample. This is consistent with the picture of regiorandom P3HT films in literature.^21^ The *I*_1460_/*I*_1520_ ratio of the point spectra in Figure 1d is around 1.3, with this larger value indicating shorter conjugated polymer segments on average in comparison to the semicrystalline sample. However, one nano‐FTIR point spectrum (plotted in orange) in Figure 1d shows clearly different form in comparison to the others, and a *I*_1460_/*I*_1520_ ratio of 1.1 indicating a greater average conjugation length. This indicates that areas with stronger molecular ordering are nonetheless present in the regiorandom film, an effect that has been previously observed.^21^ Evidence of localized ordering in the amorphous film is also present in narrow‐field SE spectra, as discussed further below in relation to Figure 3c.

Combining localized SE spectra with the results acquired with nano‐FTIR, localized variation in SE spectroscopy appears to correlate with localized variation in molecular ordering. This strengthens the correlation we find between peaks 1 and 3 in the P3HT SE spectrum and crystalline content in the film. In order to exploit this effect for local crystallinity mapping we first consider the effect of the electron beam exposure on the crystallinity.

### 2.4 Electron Beam Dose Effects on Secondary Electron Spectrum

**Figure 2:**
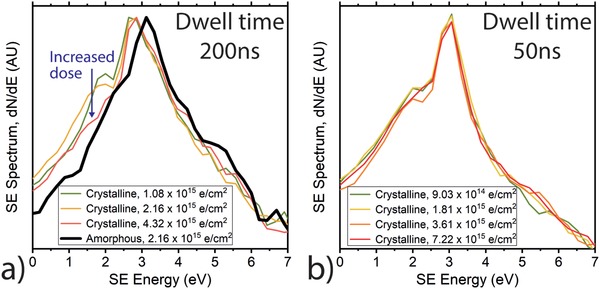
Effect of electron dose on the experimentally measured SE spectrum, from ≈10 µm^2^ areas. a) Effect of increasing dose with 200 ns dwell time and increasing line integrations. b) Effect of increasing dose with 50 ns dwell time and increasing frame integrations.

P3HT films are well known to be susceptible to electron beam damage,^25^, ^26^ with loss of molecular ordering being one of the damage effects occurring at lowest dose.^25^ In **Figure** **2**, we consider how the measured P3HT SE spectrum is affected by the electron dose and dose rate.

In Figure 2a, we present the effect of increasing electron dose on the SE spectrum measured from ≈10 µm^2^ areas of a semicrystalline P3HT sample in the Helios SEM. Up to at least an acquisition dose of 2.16 × 10^15^ electrons cm^−2^, the characteristic low‐energy peak demonstrated by semicrystalline P3HT films (peak 1 in Figure 1f) is visible. However, for a spectrum acquisition dose of 4.32 × 10^15^ electrons cm^−2^, this peak is observed to have decreased significantly in intensity, such that the spectrum appears to resemble that of an amorphous P3HT film (black plot, Figure 2a). We found the SE spectrum of the amorphous P3HT sample however appears to be largely unchanged for electron doses up to 5 × 10^15^ electrons cm^−2^.

A recent study^26^ demonstrated that loss of molecular ordering in P3HT occurred with critical electron doses of <2 × 10^15^ electrons cm^−2^ (albeit in the transmission electron microscope (TEM)). Therefore, we suggest that the degradation of the low‐energy peak in Figure 2a with increasing dose is related to the destruction of molecular ordering in the film. This further strengthens the link between peak 1 in Figure 1a,f and the crystalline content of the film.

Comparing the SE spectra in Figure 2a,b shows the importance of not only electron beam dose in SE spectrum acquisition, but also the pattern in which this dose is delivered. In Figure 2a, electron beam parameters were specifically selected to demonstrate beam damage effects (Supporting Information). In Figure 2b, we used the same electron beam scan pattern to that used to acquire the spectra in Figure 1c,f: a short 50 ns dwell time coupled with frame‐integration (each pixel was irradiated in 50 ns pulses, with a ≈20 ms delay between successive irradiation events). For both parts of Figure 2, an identical electron beam current was used.

In Figure 2b, the 7.22 × 10^15^ electrons cm^−2^ spectrum acquired with short dwell‐time and frame integration retains the clear low‐energy peak as expected from a semicrystalline sample. This contrasts the 4.32 × 10^15^ electrons cm^−2^ dose spectrum in Figure 2a, where, when measured with a lower dose but longer dwell time, the SE spectrum suggests sample damage. We expect that the short dwell time employed in Figure 2b minimizes the number of electrons excited by a single “pulse” of electron beam irradiation, and the long delay between subsequent irradiation events in the frame integration scan pattern allows time for excited electrons to relax to a lower‐energy state before repeated exposure occurs. This minimizes chemical damage to the sample.^27^, ^28^

Figure 2a suggests that SEHI techniques may have limitations when studying some highly beam‐sensitive samples, for which spectrum acquisition doses may exceed a critical dose for loss of molecular order. Similar effects are observed when studying beam‐sensitive materials with many analytical electron microscopy techniques.^29^ Understanding and accounting for potential beam damage effects is therefore crucial to any reliable SEHI study. Yet, electron dose and dose rate induced spectral changes can be used to assess whether an image obtained with a particular dose and dose rate are representative of the original material or of heavily beam damaged material.

For P3HT, Figure 2b shows that beam damage effects can be mitigated using advanced beam scanning methods, allowing localized SE spectrum acquisition from areas ≈60 nm across. The electron dose effect on the P3HT spectrum further supports the link between peak 1 (as defined in Figure 1) and the presence of crystalline material in the measurement area. Based upon this link, we apply SE hyperspectral imaging to map molecular ordering in P3HT, using machine‐learning powered techniques.

### 2.5 Secondary Electron Hyperspectral Imaging with Machine Learning: Mapping Molecular Ordering at the Surface

We used techniques demonstrated above to produce SE hyperspectral maps of P3HT samples. Non‐negative matrix factorization (NMF) techniques were used to determine the principal components causing variation in the SE spectrum across the surface of our samples. These techniques were applied using the Hyperspy software package to SEHI maps of multiple P3HT samples, as described fully in the Supporting Information.

**Figure 3:**
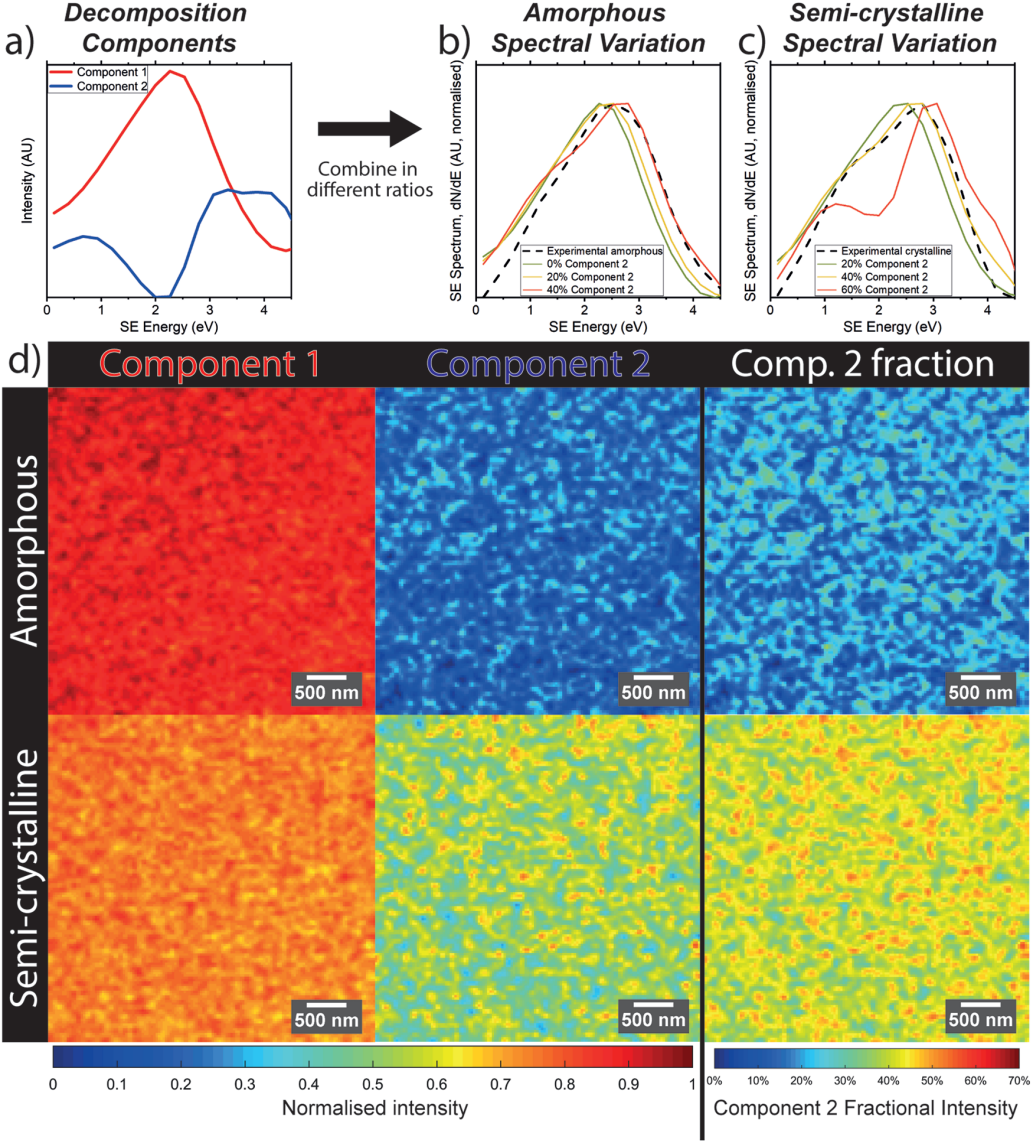
SEHI study of amorphous and semicrystalline P3HT with NMF spectral decomposition. a) Shows the two major components factorized from P3HT SE spectra. b,c) Show how these components form localized SE spectral variation when combined in different fractions, as compared to experimental spectra measured from amorphous and semicrystalline samples. d) Shows the spatially resolved relative loadings of these two spectral components across the sample surface of amorphous and semicrystalline P3HT films, as well as a map of the spatially resolved relative intensity of component 2 as a fraction of total spectrum intensity

In **Figure** **3**a, we show the two dominant spectrum factors output by the NMF decomposition algorithm. The two factors can be closely related to SE spectral features emitted from crystalline and amorphous phases. component 1 (in red) is a broad, featureless peak resembling the SE spectrum measured from amorphous P3HT in the Helios SEM (Figure 1c), with peak position at a similar energy to peak 2 as defined in Figure 1c,f. component 2 (in blue) does not resemble a SE spectrum when considered independently. However, component 2 demonstrates peaks at ≈1 and ≈4 eV, in positions similar to those of peaks 1 and 3 (as defined in Figure 1c,f), which we have linked to the presence of molecular ordering in this work. We therefore model the spectral decomposition results as reflecting the spectrum of an amorphous matrix (represented by component 1), having additional peaks when crystalline material is present (represented by convoluting components 1 and 2)

In Figure 3b, we combine components 1 and 2 in different ratios, to model the appearance of a SE spectrum consisting of different relative loadings of components 1 and 2. For low relative component 2 loadings, the modeled spectrum resembles the experimental spectrum measured from the amorphous P3HT film. The decomposition model best matches the amorphous P3HT spectrum with around ≈20% component 2 loading, with a small shoulder around the position of peak 1 (as defined in Figure 1c). Further, we observe that, for small component 2 loadings, varying the relative loading of component 2 in the modeled spectrum produces only small variation in the SE spectrum. This reflects the small degree of variation in the narrow‐field SE spectra of this sample (Figure 1c). With higher component 2 loadings, peaks 1 and 3 (as defined in Figure 1c,f) become more prominent; we observed the same effect in experimental spectra measured from samples containing higher crystallinity. This is demonstrated in Figure 3c. At component 2 loadings of ≈40%, the modeled spectrum is an excellent match to the experimental SE spectrum of the semicrystalline sample as measured in the Helios SEM (Figure 1f).

In Figure 3d, we map the relative strength of components 1 and 2 across amorphous and semicrystalline P3HT samples. Component 1 demonstrates strong loadings across both P3HT samples. For the amorphous sample, the component 1 loading is high for the large majority of the image (mean loading of ≈90%, 97% of the map is above ≈80% loading), reflecting the strong link between component 1 and amorphous material. In the semicrystalline sample, a lower average component 1 loading of ≈75% is present. This reflects the presence of significant crystalline phases alongside some highly amorphous regions up to ≈100 nm across (≈15% of the map has loading above 80%). Both amorphous and semicrystalline samples show similar standard deviation in component 1 loading, around 4%. This suggests that the amorphous component is fairly consistent for both materials. Regarding component 2, we find the semicrystalline sample to have a mean loading of ≈60% with a standard deviation of ≈11% across the SEHI map. This compares with the amorphous sample, having a mean loading of ≈20% with standard deviation around 9%. This reflects a more amorphous and homogenous sample and is expected; however, the difference in standard deviation with respect to the semicrystalline sample (11%) is small. This is most likely because component 2 variation has little effect on the shape of the spectrum at the low loadings found in the amorphous sample (effect in Figure 3b), which introduces greater error in the spectral decomposition algorithm.

To visualize SE spectral variation more accurately, the relative fraction of component 2 is also mapped in Figure 3d. Here, where *I_n_* is the localized intensity of NMF component *n*, the fractional intensity of component 2 is given by *I*_2_/(*I*_1_ + *I*_2_). As such, the component 2 fractional intensity maps eliminate any localized variation in overall spectrum intensity, possibly influenced by localized topography or charging, and instead reflect only the shape of the localized spectrum. The localized spectrum shape can be defined entirely by the relative fraction of component 2 in a NMF‐modeled spectrum, as demonstrated in Figure 3b,c.

For the semicrystalline sample, we show that the fractional loading of component 2 varies considerably between ≈25% and 60% across the semicrystalline sample. As demonstrated in Figure 3c, larger average component 2 loadings give rise to peaks 1 and 3 as denoted in Figure 1. Figure 3d suggests that while every 60 nm pixel in the SEHI map of the semicrystalline film contains some degree of crystallinity, the relative fraction of localized crystallinity varies considerably across the sample. This is in agreement with literature, where molecular‐scale aggregates and larger‐scale crystallites have been shown to co‐exist with amorphous phases.^17^ Highly ordered phases in the component 2 fraction map in Figure 3d measure around 100–200 nm in size, and are closely intermixed with more amorphous phases, as expected from more recent models of the P3HT system.^17^ The length‐scale matches well with atomic force microscope maps of similar P3HT films,^30^ as well as some P3HT structures within similarly processed blend films that we have previously imaged with a higher‐resolution SEM technique.^4^ It is important to note however that the morphology of P3HT is hierarchical,^31^ and in Figure 3 we only map morphology on a length scale limited by resolution due to electron dose with the current experimental setup.

In the fractional component 2 map of the amorphous sample, we observe that the regiorandom amorphous sample shows various regions containing up to ≈30% or even greater fractional loading of component 2. The presence of some regions of higher component 2 loading indicates some limited, localized molecular ordering in a sample that is often depicted as uniformly amorphous. This effect was previously detected,^21^ with small regioregular segments even in a “perfect” regiorandom molecule enabling the formation of small crystallites. These are small enough to be “invisible” to many characterization techniques such as wide‐angle X‐ray scattering;^21^ however, our localized SE spectra suggest at their presence here. We detect areas 200–300 nm across containing a component 2 (crystalline) fraction >20%, alongside similar‐sized areas having negligible component 2 loading (i.e., almost entirely amorphous).

Figure 3 depicts a technique with strong potential for mapping localized sample properties in a rapid, easily implemented way. The acquisition of a full SEHI map takes just ≈2 min using an automated acquisition script on an unmodified SEM. However, the spatial and energy resolution of this technique is limited by electron dose effects. Image magnification (i.e., pixel resolution) and acquisition time (i.e., signal‐to‐noise ratio) were limited in order to acquire the SEHI maps in Figure 3d without incurring the beam damage effects presented in Figure 2. It is known, for example, that smaller crystalline structure than that visible in Figure 3 is present in P3HT films.^32^ We expect that this dose‐limited resolution could be improved significantly with advances in hardware, for example with more advanced SE spectrometers.^1^

Figure 3 demonstrates how the links between molecular order and SE spectral features suggested in Figures 1 and 2 may be employed to map material properties. However, in order to apply the technique more broadly and confidently, we investigate the fundamental origin of these SE spectral features more closely from a theoretical standpoint.

### 2.6 Monte Carlo Modeling of the SE Spectrum

We simulated the shape of the P3HT SE spectrum from a Monte Carlo model of the physical processes and interactions resulting from a primary electron beam impinging on a P3HT film. An overview of the modeling methods used is included in the Supporting Information to this work; however, the main input parameters are the complex dielectric function, and the electron affinity (χ). As the electron affinity is closely linked to electronic dipoles at the surface, and hence to molecular order and orientation,^33^ below we present the results, showing the effect of different electron affinities on the spectra.

**Figure 4:**
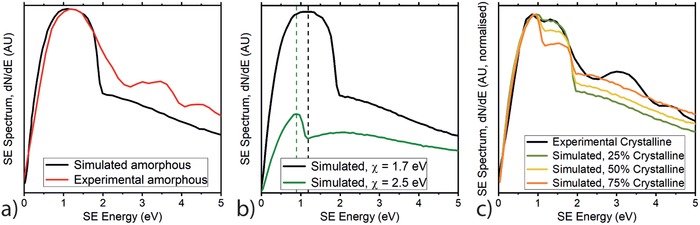
Monte Carlo modeling of secondary electron spectra. a) Comparison of experimental and simulated spectra for amorphous P3HT. b) Effect of changing the electron affinity parameter in Monte Carlo simulation of SE spectrum. c) Simulating the SE spectrum of a semicrystalline P3HT film by considering the different electronic properties of amorphous and crystalline phases.

The accuracy of the simulation for this work was verified by modeling the SE spectrum of an amorphous P3HT film (**Figure** **4**a). Amorphous P3HT is an ideal test case for this purpose; electron transport in an amorphous film is simpler to model due to its relative homogeneity.^2^ We compared the modeled spectra with experimental spectra measured in the FEI Sirion tool (Figure 1a). The modeled spectrum demonstrates two primary features: a large, dominant peak at low energies (<2 eV), with a long tail at higher energies. This produces a good match for the general shape of the experimental spectrum.

In Figure 4b, we explore the effect of electron affinity on the SE spectrum shape. The values of P3HT electron affinity in literature take a range of values, from 2.1^14^ to 3.2 eV,^34^ as such the χ values modeled in Figure 4b (χ = 1.7 and 2.5 eV) are a reasonable match to real P3HT films. We observe that with a higher electron affinity value, the SE spectrum drops considerably in overall intensity, and the low‐energy peak becomes narrower and less intense relative to the tail. This reflects electron affinity as an analogue for the energy barrier that an internal SE must overcome if it is to be emitted. A larger electron affinity therefore has the effect “cutting‐off” low‐energy SEs from the emitted spectrum, and “compressing” the low‐energy peak in to a smaller energy range. At higher electron affinities, the higher‐energy spectral feature that is observed as a tail for χ = 1.7 eV is revealed to be a peak at ≈2 eV in the χ = 2.5 eV spectrum. The energy position of this feature appears independent of the electron affinity value, suggesting it may be representative of some other feature of the sample.

In Figure 4c, the effect of different electron affinity values on the SE spectrum shape is used to explain the “double” peak feature observed around 1 eV in the wide‐field SE spectrum of semicrystalline P3HT (Figure 1a). We model the semicrystalline P3HT film as a two‐phase system consisting of domains of different electron affinity. A SE can then be emitted from an area of the film with one of two electron affinity values. By setting the electron affinities of these two phases to χ = 1.7 and 2.5 eV respectively, our modeled P3HT spectrum accurately recreates the double‐peak feature around 1 eV (peaks 1 and 2 in Figure 1a). As the semicrystalline P3HT sample emits a SE peak at lower energies than the amorphous sample in all of our experimental SE spectra (Figures 1 and 2), we ascribe an average χ = 2.5 eV to the crystalline phase, and an average χ = 1.7 eV to the amorphous phase. P3HT films with a greater degree of molecular ordering have a larger bulk electron affinity in literature.^33^ The difference in the electron affinity we model from crystalline and amorphous domains relates to localized variation that is typically averaged by conventional bulk measurements of χ.

The relative intensities of the two low‐energy peaks in Figure 4c can be used to infer the fraction of the sample surface consisting of each domain. Matching the semicrystalline SE spectrum in Figure 1a to this data, we approximate from our data that ≈25% of the semicrystalline P3HT film surface is crystalline. The absolute crystallinity content of P3HT films is difficult to compare with literature due to the effects of surfaces and interfaces on the absolute values.^21^ However we note that this crystallinity value is significantly smaller than that measured from bulk samples of comparable molecular weight and regioregularity (≈50%) using X‐ray scattering and nuclear magnetic resonance methods.^21^ This is expected for a spin‐cast film.^21^ We also investigated the role of differences in the complex dielectric function but find a very weak influence (see Supporting Information)

Our findings here are an important demonstration that bulk electronic measurements perhaps generate a simplified picture of a nanostructured film, where crystalline and amorphous phases demonstrate significantly different electronic properties.^17^ As electronic devices shrink to ever smaller sizes, considering these nanoscale variations will be of increasing importance.

## 3 Conclusion

In this work, we have delivered important advancements in SE spectroscopy and SEHI, both building upon and underpinning our previous work to showcase a characterization technique with real potential. We demonstrated that specific spectral features correlate with crystalline content in the film, through both empirical study of experimental spectra and from a theoretical standpoint with a Monte Carlo modeling technique. Further, by comparing our SE spectroscopy results with a study of nanoscale chemistry through nano‐FTIR, we showed that localized variation in P3HT SE spectra matches well with localized variation in conjugation length, an indicator of molecular order.

By modeling the P3HT SE spectrum, we showed that the spectral features can be related to the localized electronic properties of the film. These electronic properties are themselves associated with localized molecular ordering. We found that the electron affinity of the sample is a dominant factor determining the shape of the spectrum.

Finally, we demonstrated SEHI, combined with data analysis powered by machine learning, as a tool for mapping localized molecular order in P3HT with 60 nm^2^ pixel size. Our SEHI techniques can be applied on various modern SEM systems with no hardware modification, with data acquisition performed in ≈2 min and no extraordinary sample preparation required.

We anticipate that in time, advancements in detector hardware will enable even higher resolution SEHI maps of beam sensitive materials. In the meantime, relevant implementations of low‐dose TEM methods, such as advanced denoising or compressed sensing,^35^, ^36^ may enable further progress. We expect that with further advancements such as these, SE energy spectroscopy and SEHI techniques can develop in to standalone, powerful, and versatile materials characterization tools that exploit the power of the modern‐day SEM to its fullest extent.

## Conflict of Interest

The authors declare no conflict of interest.

## Supporting information

Supplementary
