# Supplementary material for: Mapping Polymer Molecular Order in the SEM with Secondary Electron Hyperspectral Imaging
Source: Adv Sci (Weinh). 2019 Jan 20;6(5):1801752. doi: 10.1002/advs.201801752 (PMC6402282; doi:10.1002/advs.201801752)
Supplement: Supplementary file 1 — Supplementary [file ADVS-6-1801752-s001.pdf]

Copyright WILEY-VCH Verlag GmbH & Co. KGaA, 69469 Weinheim, Germany, 2019.

ADVANCED SCIENCE
Open Access

Supporting Information

for *Adv. Sci.,* DOI: 10.1002/advs.201801752

Mapping Polymer Molecular Order in the SEM with Secondary Electron Hyperspectral Imaging

*Robert C. Masters, Nicola Stehling, Kerry J. Abrams, Vikas Kumar, Martina Azzolini, Nicola M. Pugno, Maurizio Dapor, Andreas Huber, Philip Schäfer, David G. Lidzey, and Cornelia Rodenburg**

Mapping polymer molecular order in the SEM with secondary electron hyperspectral imaging

Supplementary Information

1. Experimental Methods

Sample Preparation

For semi-crystalline P3HT films, P3HT was purchased from Ossila Ltd. (UK), batch M105 with Mn = 49500 and regioregularity = 95.5%, and spin-cast from a 25 mg.ml^{-1} dichlorobenzene solution at 1000 rpm to encourage crystal growth. Films were then thermally annealed at 170°C for 20 minutes. For amorphous P3HT films, regiorandom P3HT (510823) was purchased from Sigma-Aldrich, and spin-cast from a 25 mg.ml^{-1} chlorobenzene solution at 1500 rpm. All solution formulation, spin-casting and annealing processes were performed in a nitrogen glovebox environment. Films were cast on to silicon substrates, cleaned in an ultrasonic bath with Hellmanex solution and isopropanol, and finally treated in UV-ozone for 10 minutes prior to the casting process. All samples were stored under nitrogen, and transported to the electron microscope under vacuum conditions to prevent degradation of the film surface.

Electron microscopy and measurement of SE spectra

All electron microscopy work was performed with a primary beam energy E_0 = 700 eV, with a beam current around 12 pA. These parameters were optimised to minimise charging and electron beam damage issues. Samples were imaged with the through-the-lens detector with immersion-lens active. Two scanning electron microscopes were used: a FEI Sirion, an older microscope with well-documented SE spectroscopy capabilities[1], [2], and a state-of-the-art Thermo Fisher Helios NanoLab G3 UC capable of sub-nanometre imaging resolution[3] and offering an advanced level of electron beam control for dose minimisation.

For both microscopes, energy filtering of SEs was performed by altering the bias on electrodes within the through lens detector assembly. The exact mechanism of energy filtering is similar but differs slightly for each microscope (an overview of SE energy-filtering strategies is given in reference[4]). SE energy spectra and hyperspectral SE images were acquired by capturing a stack of images of the sample whilst sweeping through a range of TLD electrode bias values (again, a detailed overview is given in references[3], [4]).

For work on the FEI Sirion tool, SE spectra were acquired from 20 µm^2 areas, with each image in the spectrum stack captured in TV scan mode with 228x228 pixel resolution and 4-fold frame integration. For all localised spectrum measurements in the Helios tool (excepting Figure 2a), SE spectra were acquired from 4.1 µm^2 areas with a monochromated primary beam, with each image in the spectrum stack acquired using 210x210 pixel resolution, 50 pA dwell-time, 128-fold frame integration, and a scan-interlace of 8. For both microscopes, each SE spectrum stack consisted of 51 images. Our justification for using these parameters is discussed regarding Figure 2. For Figure 2b, the number of frame integrations was varied in order to control the spectrum acquisition dose.

To acquire the spectra in Figure 2a, we used the Helios tool with the same beam current, measurement area and resolution, but with a line-integration scan pattern and a 200ns dwell time per pixel (200ns irradiation 'pulses', with a ~0.15ms delay between successive pulses). The number of line integrations was varied in order to control the spectrum acquisition dose.

Comparison of Figure 1a with 1c,f demonstrates that the Helios SEM appears to give lower-intensity peaks at low SE energies in comparison to the FEI Sirion. This is likely a result of the limitations of the Helios SEM as a SE spectrometer, which we have investigated previously [4]

SE spectrum and SEHI data analysis

SE spectra were processed through an in-house MATLAB script to organise and process the spectrum image stack in to the spectra in Figure 1[3]. A Gaussian point spread function was applied to reduce the effect of noise in both the spatial and energy dimensions, with a standard deviation of 0.53 eV in the energy dimension and 1 pixel in the spatial dimensions.

The Hyperspy package for python[5] was used to perform the spectral decomposition presented in Figure 4. Three SEHI images of both amorphous and semi-crystalline P3HT samples were analysed together by the same decomposition algorithm, to ensure the generated components were consistent for both samples and across different areas of the same sample. Pixel binning with a factor of 3 was used for this analysis (i.e., 3x3 pixel areas were analysed as single pixels) to reduce the impact of shot noise on the analysis. Further, the integrated SE intensity of each analysed SEHI image was normalised across the full measured SE energy range prior to decomposition. Spectral decomposition was performed with a non-negative matrix factorisation (NMF) algorithm, in order to ensure all factors and loadings generated by the algorithm were physical (i.e. ≥ 0). We found that the first two components output by this algorithm excellently described the spectral variance found in the localised SE spectra of P3HT samples.

nano-FTIR

For mid-IR near-field spectroscopy, a commercial scattering-type scanning near-field optical microscope was used (neaspec GmbH, Germany). The system is based on standard AFM technology where a conventional metal-coated (Pt/Ir) tip is oscillating in intermittent contact vertically to the sample surface. The tip acts simultaneously as an AFM probe and as a near-field probe[6]. Tip and sample are illuminated via a parabolic mirror objective with a broadband mid-infrared supercontinuum laser (neaspec GmbH, max. frequency range ca. 650–2200 cm^{-1}, with average 1 mW power), which is generated by difference frequency generation. The tip-scattered light is analyzed with an asymmetric Fourier transform spectrometer where tip and sample are located in one of the interferometer arms. The detector signal is demodulated at a frequency O2 (2x AFM tip oscillation frequency) for effective background suppression. An interferogram is measured by recording the demodulated detector signal as a function of the position of the reference mirror at a fixed tip position. Subsequent Fourier transform of the recorded interferogram yields the complex-valued near-field point spectrum from the 10nm length scale volume just underneath the AFM-tip. The complex spectrum can be represented as nano-FTIR reflectivity and absorption spectra[7], [8].

Monte Carlo Modelling

A Monte Carlo method was used to simulate the shape of the secondary electron emission peak by considering a monoenergetic electron beam impinging on the investigated material. The trajectories of both primary and secondary electrons were calculated. The electrons escaping from the material were counted as a function of their kinetic energy to obtain the emission spectrum.

In particular the simulation modelled: (i) the elastic scattering between electrons and atomic nuclei, (ii) the inelastic scattering between travelling electrons and target electrons, (iii) the electron-phonon interaction and (iv) the trapping phenomenon[9]. The parameters employed are the properties of P3HT taken from literature[10] (C_{trap} = 0.001 Å-1, γ_{trap} = 0.105 eV-1). The electron-phonon interaction, which influences the low energy electron region, was treated by applying the Froehlich theory [11]. The value of high frequency and static dielectric constants, respectively equal to ε_{∞}= 3.6 and ε_0 = 3.0[12], were employed in the calculation. Moreover, the single energy loss due to the electron-phonon interaction was set to W_{ph} = 0.05 eV.

The inelastic scattering, due to electron-electron cloud interaction, was considered by implementing the Ritchie dielectric theory[13]. It involves the energy loss function, described as a sum of Drude-Lorentz functions representing electron oscillations[14]. The values of the strength, energy and width of the oscillators are those reported in Ref. [15].

The elastic scattering was evaluated starting from the elastic scattering cross section by applying the Mott theory[16]. The P3HT elastic scattering cross section was obtained by a linear combination of elastic cross sections of the single elements composing the molecule. The coefficients of the combination are given by the stoichiometry of the compound. The analytical formulation of atomic potentials by Salvat was used [17]. Moreover, the Ganachaud and Mokrani correction was applied to the total elastic scattering cross section[18] by using the parameter α equal to 0.01.

Secondary electrons are generated as a result of inelastic interactions: if the energy loss of the travelling electron is larger than the threshold energy, this energy is transferred to an electron of a target atom. Thus, the secondary electron is emitted and its trajectory is calculated in the model. The threshold energy, in this case, was set equal to 2.5 eV, a value corresponding to the mean value of energy band gaps found in the literature[19].

A key role in the calculation is represented by the electron affinity, acting as the energy barrier that electrons must overcome to be emitted. The surface of the P3HT shows amorphous and crystalline regions. To model this feature, regions with different electrons affinities were considered in the simulation.

The modelled SE spectra were compared to the experimental results acquired in the FEI Sirion SEM. The lower dose used to acquire SE spectra in the Sirion SEM means that the spectra in Figure 1a are less likely to be affected by electron beam damage or charging, effects which are not accounted for in the Monte Carlo model.

2. Modelling SE spectra: effect of plasmon energy

In Supplementary Figure 1, we explore the importance of a different modelling parameter, the intensity of π-electron plasmon oscillator, to the shape of the modelled SE spectrum (Figure 4, main work). This is a useful sample feature in the discussion of molecular ordering, as the strength of the π-plasmon has been closely related to the presence of molecular ordering in the material in electron energy loss spectrum (EELS) studies in the TEM[20], [21]. Considering SE spectroscopy, the excitation of π plasmons is a significant source of energy loss for SEs within the material[15], influencing the shape of the SE spectrum as a result[15], [22].

Our previous work[15] showed that the average π-plasmon oscillator is ~1.5 times stronger in P3HT films having a higher degree of crystallinity (localised differences may be considerably larger, however). The results in Supplementary Figure 1 show that suggest that crystalline P3HT phases with a (considerably) stronger π-electron structure would display a spectrum peak at lower energies. We expect this trend from our experimental studies in Figure 1. Supplementary Figure 1 suggests this effect is relatively small in comparison to that of electron affinity (Figures 3b-c), and as such we expect that electron affinity remains the dominant effect resulting in the strength of peak 1 in Figure 1f.

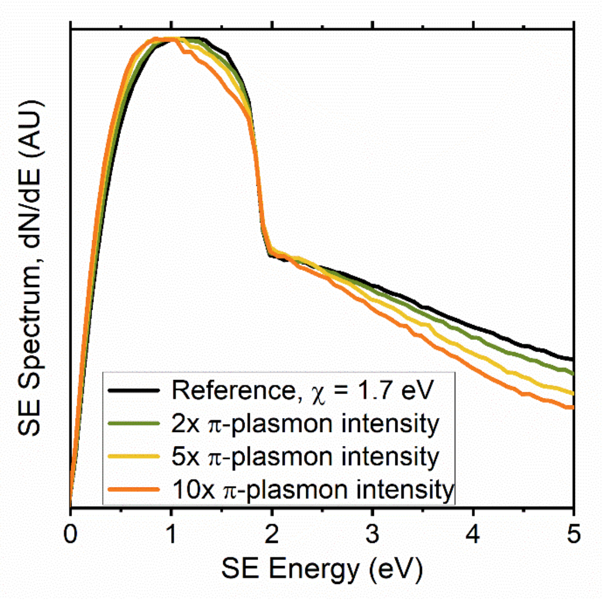

Supplementary Figure 1: *Effect of* π-plasmon intensity on the shape of the simulated P3HT SE *spectrum with* χ*=1.7eV*

3. References

[1] P. Kazemian, S. a. M. Mentink, C. Rodenburg, and C. J. Humphreys, “High resolution quantitative two-dimensional dopant mapping using energy-filtered secondary electron imaging,” J. Appl. Phys., vol. 100, no. 5, p. 54901, 2006.

[2] M. a E. Jepson, B. J. Inkson, R. Beanland, a K. W. Chee, C. J. Humphreys, and C. Rodenburg, “Progress towards site-specific dopant profiling in the scanning electron microscope,” J. Phys. Conf. Ser., vol. 209, p. 12068, Feb. 2010.

[3] R. C. Masters, A. J. Pearson, T. S. Glen, F.-C. Sasam, L. Li, M. Dapor, A. M. Donald, D. G. Lidzey, and C. Rodenburg, “Sub-nanometre resolution imaging of polymer–fullerene photovoltaic blends using energy-filtered scanning electron microscopy,” Nat. Commun., vol. 6, p. 6928, 2015.

[4] N. Stehling, R. Masters, Y. Zhou, R. O’Connell, C. Holland, H. Zhang, and C. Rodenburg, “New perspectives on nano-engineering by secondary electron spectroscopy in the helium ion and scanning electron microscope,” MRS Commun., vol. 8, no. 2, pp. 226–240, Jun. 2018.

[5] F. de la Peña, T. Ostasevicius, V. T. Fauske, P. Burdet, E. Prestat, P. Jokubauskas, M. Nord, M. Sarahan, K. E. MacArthur, D. N. Johnstone, J. Taillon, J. Caron, V. Migunov, T. Furnival, A. Eljarrat, S. Mazzucco, T. Aarholt, M. Walls, T. Slater, F. Winkler, B. Martinea, G. Donval, R. McLeod, E. R. Hoglund, I. Alxneit, I. Hjorth, T. Henninen, L. F. Zagonel, A. Garmannslund, and 5ht2, “Hyperspy 1.3.2.” Zenodo, 2018.

[6] F. Keilmann and R. Hillenbrand, “Near-field microscopy by elastic light scattering from a tip,” Philos. Trans. R. Soc. A Math. Phys. Eng. Sci., vol. 362, no. 1817, pp. 787–805, Apr. 2004.

[7] F. Huth, A. Govyadinov, S. Amarie, W. Nuansing, F. Keilmann, and R. Hillenbrand, “Nano-FTIR Absorption Spectroscopy of Molecular Fingerprints at 20 nm Spatial Resolution,” Nano Lett., vol. 12, no. 8, pp. 3973–3978, Aug. 2012.

[8] I. Amenabar, S. Poly, M. Goikoetxea, W. Nuansing, P. Lasch, and R. Hillenbrand, “Hyperspectral infrared nanoimaging of organic samples based on Fourier transform infrared nanospectroscopy,” Nat. Commun., vol. 8, 2017.

[9] J. P. Ganachaud and A. Mokrani, “Theoretical study of the secondary electron emission of insulating targets,” Surf. Sci., vol. 334, pp. 329–341, 1995.

[10] J. Schafferhans, A. Baumann, A. Wagenpfahl, C. Deibel, and V. Dyakonov, “Oxygen doping of P3HT:PCBM blends: Influence on trap states, charge carrier mobility and solar cell performance,” Org. Electron., vol. 11, no. 10, pp. 1693–1700, 2010.

[11] H. Fröhlich, “Electrons in lattice fields,” Adv. Phys., vol. 3, pp. 325–361, 1954.

[12] R. Singh, R. K. Singh, J. Kumar, R. Kant, and V. Kumar, “The origin of DC electrical conduction and dielectric relaxation in pristine and doped poly(3-hexylthiophene) films,” J. Polym. Sci. Part B Polym. Phys., vol. 48, no. 10, pp. 1047–1053, May 2010.

[13] R. H. Ritchie and A. Howie, “Electron excitation and the optical potential in electron microscopy,” Philos. Mag., vol. 36, no. 2, pp. 463–481, Aug. 1977.

[14] M. Azzolini, T. Morresi, G. Garberoglio, L. Calliari, N. M. Pugno, S. Taioli, and M. Dapor, “Monte Carlo simulations of measured electron energy-loss spectra of diamond and graphite: Role of dielectric-response models,” Carbon N. Y., vol. 118, pp. 299–309, 2017.

[15] M. Dapor, R. C. Masters, I. Ross, D. G. Lidzey, A. J. Pearson, I. Abril, R. Garcia-Molina, J. Sharp, M. Unčovský, T. Vystavel, F. Mika, and C. Rodenburg, “‘Secondary electron spectra of semi-crystalline polymers – A novel polymer characterisation tool?,’” J. Electron Spectros. Relat. Phenomena, vol. 222, pp. 1–11, Jan. 2017.

[16] N. F. Mott, “The Scattering of Fast Electrons by Atomic Nuclei,” Proc. R. Soc. A Math. Phys. Eng. Sci., vol. 124, no. 794, pp. 425–442, 1929.

[17] F. Salvat, J. D. Marťnez, R. Mayol, and J. Parellada, “Analytical Dirac-Hartree-Fock-Slater screening function for atoms (Z=1–92),” Phys. Rev. A, vol. 36, no. 2, pp. 467–474, Jul. 1987.

[18] M. Azzolini, T. Morresi, K. Abrams, R. Masters, N. Stehling, C. Rodenburg, N. M. Pugno, S. Taioli, and M. Dapor, “Anisotropic Approach for Simulating Electron Transport in Layered Materials: Computational and Experimental Study of Highly Oriented Pyrolitic Graphite,” J. Phys. Chem. C, vol. 122, no. 18, pp. 10159–10166, May 2018.

[19] K. Kanai, T. Miyazaki, H. Suzuki, M. Inaba, Y. Ouchi, and K. Seki, “Effect of annealing on the electronic structure of poly(3-hexylthiophene) thin film.,” Phys. Chem. Chem. Phys., vol. 12, no. 1, pp. 273–282, 2010.

[20] H. Ahn, D. W. Oblas, and J. E. Whitten, “Electron Irradiation of Poly(3-hexylthiophene) Films,” Macromolecules, vol. 37, no. 9, pp. 3381–3387, May 2004.

[21] M. Pfannmöller, H. Flügge, G. Benner, I. Wacker, C. Sommer, M. Hanselmann, S. Schmale, H. Schmidt, F. a Hamprecht, T. Rabe, W. Kowalsky, and R. R. Schröder, “Visualizing a homogeneous blend in bulk heterojunction polymer solar cells by analytical electron microscopy.,” Nano Lett., vol. 11, no. 8, pp. 3099–3107, Aug. 2011.

[22] M. Dapor, Transport of Energetic Electrons in Solids. Berlin: Springer, 2014.
